# Grain Intake and Cardiometabolic Health—Towards Precision Nutrition

**DOI:** 10.3390/nu15214605

**Published:** 2023-10-30

**Authors:** Xin Liu, Lin Shi, Liang Sun

**Affiliations:** 1 Key Laboratory for Disease Prevention and Control and Health Promotion of Shaanxi Province, Department of Epidemiology and Biostatistics, School of Public Health, Global Health Institute, Xi’an Jiaotong University Health Science Center, Xi’an 710061, China; 2 School of Food Engineering and Nutritional Science, Shaanxi Normal University, Xi’an 710119, China; linshi198808@snnu.edu.cn; 3 Ministry of Education Key Laboratory of Public Health Safety, School of Public Health, Institute of Nutrition, Fudan University, Shanghai 200032, China; sun_liang@fudan.edu.cn

Grains are widely consumed all over the world, providing calories, macronutrients, micronutrients, dietary fiber, minerals, and plenty of phytochemicals [1]. These nutritional factors are essential for the homeostasis of the human metabolism and multiple organ functions [1]. However, global dietary patterns are complex, and are comprehensively influenced by social/economic development and differences in regional food cultures. Although it has been consistently suggested through the form of meta-analysis that a higher whole grain intake is associated with lower risks of type 2 diabetes [2], cardiovascular disease, and total mortality [3], and that refined grains, such as white rice, have the opposite story [4], evidence that takes into account different grain categories and cooking methods is still lacking. A typical example could be that popcorn intake has been positively associated with type 2 diabetes [5] and coronary heart disease [6] based on prospective studies in the United States. Clearly, the relatively higher glycemic index [7] and trans fat content [8] of popcorn merit caution. Another concern might be that porridges containing certain whole grains, such as millet, may have a high glycemic index with little amounts of added whole grain; this may have skewed certain epidemiological results [9]. Meanwhile, people with special health conditions may have altered dietary habits and gut microbiota profiles; thus, diet–cardiometabolic health relations may differ between sub-populations. For instance, in constipation patients, we identified that the intake of dried beans, but not the intake of other whole grains, was linked with a lower hypertension risk [10]. It should be noted that beans are traditionally consumed together with other grains, and are considered to be a major coarse grain in Chinese culture [11]. Therefore, more evidence that supports such beneficial effects is needed to convey more precise recommendations regarding specific grain categories, cooking methods, daily intake amounts, health conditions, etc.

Recently, high-throughput omics techniques have enabled the comprehensive understanding of the biological perturbations that are associated with dietary intake and other environmental exposures [12,13]. Alterations in the circulating metabolites and gut microbiota species in response to different grain consumptions have been reported. These findings may shed fresh light upon the mechanism of cardiovascular benefits upon interventions involving grain consumption. For example, in 50 Danish adults, a whole-grain intervention of 179 g/day for 8 weeks substantially reduced their body weight, serum interleukin (IL)-6, and C-reactive protein levels, but no major change was observed for their fecal microbiomes [14]. However, in this Special Issue, an N-of-1 trial showed that a short intervention of 6 days on the proportion of carbohydrate intake may result in persistent changes to the gut mycobiome, closely linked with glycemic metrics after a high- or low-carbohydrate intervention [15]. Moreover, in 62 Chinese participants with mild hypercholesterolemia, an oat intake of 80 g/day for 45 days reduced their LDL and total cholesterol levels significantly, which was accompanied by metabolite changes including sphingosine and phosphatidylcholine, suggesting relative pathways [16]. In a recent intervention study with high or low levels of resistant starch wheat incorporated into diets among healthy adults, the abundances of fecal butyrate and SCFA-producing bacteria were altered, which are associated with gastrointestinal health [17]. Taken together, multiple omics techniques have advanced our understanding of the beneficial effects of dietary changes, such as modified grain consumptions, but evidence for specific grains, unique health conditions, and long-term interventions is still needed.

The precise prediction of individual responses to nutritional interventions could function as fundamental scientific evidence towards the practice of precision nutrition in clinical and community settings. For example, various genotypes were found to interact with different carbohydrate intake levels in relation to weight/fat loss and other cardiovascular traits in the POUNDs Lost trial [18]. We also found that baseline untargeted metabolomic profiling was predictive of a favorable body composition change in response to conjugated linoleic acid supplementation [19]. Machine learning approaches may also enhance the identification of predictive baseline metabolomic or gut microbiota traits in relation to intervention responsiveness [20]. For intervention studies involving grains, it has been reported that the abundance of an individual gut microbial species, namely, *Prevotella*, was able to predict weight loss in a 6-week whole-grain intervention [21]. Clearly, there is great potential in incorporating multi-omics approaches in the early prediction of grain intervention responsiveness for evidence towards precision nutrition.

The majority of the world’s population consume a large amount of grains every year, which contributes to the greatest proportion of calories in our diet. There is a requirement for more detailed research outcomes on the health benefits of different grains and their underlying mechanisms; attempts must be made to connect genetic and non-genetic susceptibilities with the optimization of our health, driven by the intake of grains.

## References

[1] Zhang Y., Capanoglu E., Jiao L., Yin L., Liu X., Wang R., Xiao J., Lu B. (2022). Coarse cereals modulating chronic low-grade inflammation: Review. Crit. Rev. Food Sci. Nutr..

[2] Reynolds A.N., Akerman A.P., Mann J. (2020). Dietary fibre and whole grains in diabetes management: Systematic review and meta-analyses. PLoS Med..

[3] Aune D., Keum N., Giovannucci E., Fadnes L.T., Boffetta P., Greenwood D.C., Tonstad S., Vatten L.J., Riboli E., Norat T. (2016). Whole grain consumption and risk of cardiovascular disease, cancer, and all cause and cause specific mortality: Systematic review and dose-response meta-analysis of prospective studies. BMJ.

[4] Hu E.A., Pan A., Malik V., Sun Q. (2012). White rice consumption and risk of type 2 diabetes: Meta-analysis and systematic review. BMJ.

[5] Hu Y., Ding M., Sampson L., Willett W.C., Manson J.E., Wang M., Rosner B., Hu F.B., Sun Q. (2020). Intake of whole grain foods and risk of type 2 diabetes: Results from three prospective cohort studies. BMJ.

[6] Hu Y., Willett W.C., Manson J.A.E., Rosner B., Hu F.B., Sun Q. (2022). Intake of whole grain foods and risk of coronary heart disease in US men and women. BMC Med..

[7] Foster-Powell K., Holt S.H., Brand-Miller J.C. (2002). International table of glycemic index and glycemic load values: 2002. Am. J. Clin. Nutr..

[8] Wang Q., Imamura F., Ma W., Wang M., Lemaitre R.N., King I.B., Song X., Biggs M.L., Delaney J.A., Mukamal K.J. (2015). Circulating and dietary trans fatty acids and incident type 2 diabetes in older adults: The Cardiovascular Health Study. Diabetes Care.

[9] Almaski A., Shelly C., Lightowler H., Thondre S. (2019). Millet intake and risk factors of type 2 diabetes: A systematic review. J. Food Nutr. Disor..

[10] Li J., Ma G., Xie J., Xu K., Lai H., Li Y., He Y., Yu H., Liao X., Wang X. (2023). Differential gut microbiota, dietary intakes in constipation patients with or without hypertension. Mol. Nutr. Food Res..

[11] Liu X., Lai H., Mi B., Qi X., Gan W., Du H. (2020). Associations of Coarse Grain Intake with Undiagnosed Hypertension among Chinese Adults: Results from the China Kadoorie Biobank. Nutrients.

[12] Palmnäs M., Brunius C., Shi L., Rostgaard-Hansen A., Torres N.E., González-Domínguez R., Zamora-Ros R., Ye Y.L., Halkjær J., Tjønneland A. (2020). Perspective: Metabotyping—A potential personalized nutrition strategy for precision prevention of cardiometabolic disease. Adv. Nutr..

[13] Yang M., Yan T., Yu M., Kang J., Gao R., Wang P., Zhang Y., Zhang H., Shi L. (2020). Advances in understanding of health-promoting benefits of medicine and food homology using analysis of gut microbiota and metabolomics. Food Front..

[14] Roager H.M., Vogt J.K., Kristensen M., Hansen L.B.S., Ibrügger S., Mærkedahl R.B., Bahl M.I., Lind M.V., Nielsen R.L., Frøkiær H. (2019). Whole grain-rich diet reduces body weight and systemic low-grade inflammation without inducing major changes of the gut microbiome: A randomised cross-over trial. Gut.

[15] Tian Y.Y., Gou W.L., Ma Y., Shuai M.L., Liang X.X., Fu Y.Q., Zheng J.S. (2023). The Short-Term Variation of Human Gut Mycobiome in Response to Dietary Intervention of Different Macronutrient Distributions. Nutrients.

[16] Xu D., Wang S., Feng M., Shete V., Chu Y., Kamil A., Yang C., Liu H., Xia H., Wang X. (2021). Serum Metabolomics Reveals Underlying Mechanisms of Cholesterol-Lowering Effects of Oat Consumption: A Randomized Controlled Trial in a Mildly Hypercholesterolemic Population. Mol. Nutr. Food Res..

[17] Gondalia S.V., Wymond B., Benassi-Evans B., Berbezy P., Bird A.R., Belobrajdic D.P. (2022). Substitution of Refined Conventional Wheat Flour with Wheat High in Resistant Starch Modulates the Intestinal Microbiota and Fecal Metabolites in Healthy Adults: A Randomized, Controlled Trial. J. Nutr..

[18] Qi L., Heianza Y., Li X., Sacks F.M., Bray G.A. (2023). Toward Precision Weight-Loss Dietary Interventions: Findings from the POUNDS Lost Trial. Nutrients.

[19] He Y.F., Xu K., Li Y.F., Chang H., Liao X., Yu H., Tian T., Li C., Shen Y., Wu Q. (2022). Metabolomic Changes Upon Conjugated Linoleic Acid Supplementation and Predictions of Body Composition Responsiveness. J. Clin. Endocr. Metab..

[20] Kirk D., Kok E., Tufano M., Tekinerdogan B., Feskens E.J.M., Camps G. (2022). Machine Learning in Nutrition Research. Adv. Nutr..

[21] Christensen L., Vuholm S., Roager H.M., Nielsen D.S., Krych L., Kristensen M., Astrup A., Hjorth M.F. (2019). Prevotella Abundance Predicts Weight Loss Success in Healthy, Overweight Adults Consuming a Whole-Grain Diet Ad Libitum: A Post Hoc Analysis of a 6-Wk Randomized Controlled Trial. J. Nutr..

